# Current corticosteroid therapeutic strategy for community-acquired pneumonia in adults: indications, dosage, and timing

**DOI:** 10.1186/s40560-025-00809-8

**Published:** 2025-07-01

**Authors:** Seitaro Fujishima

**Affiliations:** Center for Preventive Medicine, Keio University School of Medicine, 1-3-1 Azabudai, Minato-ku, Tokyo, 106-0041 Japan

**Keywords:** Community-acquired pneumonia, Guidelines, COVID-19, Glucocorticoids, Mineralocorticoids, Dexamethasone, Sepsis, Septic shock, Acute respiratory distress syndrome

## Abstract

Despite advances in treatment and the expansion of standard care, pneumonia remains a major cause of mortality. It frequently leads to complications such as septic shock and acute respiratory distress syndrome (ARDS), both of which carry high fatality rates. Although antimicrobial therapy is the cornerstone of treatment, additional supportive care and adjunctive therapies, such as corticosteroids, are often required, especially in severe community-acquired pneumonia (CAP).

Recent updates to major guidelines on CAP, sepsis, ARDS, and critical illness-related corticosteroid insufficiency generally support corticosteroid use in severe CAP. However, the REMAP-CAP randomized controlled trial, published in 2025, failed to demonstrate significant benefit, potentially influencing future recommendations. Currently, corticosteroid therapy should be individualized based on CAP severity, particularly the degree of hypoxemia and respiratory failure. In eligible patients, early initiation and flexible duration of corticosteroid use based on clinical response may be appropriate. For nonbacterial pneumonia, strong evidence supporting corticosteroid use exists only for COVID-19 and *Pneumocystis jirovecii* pneumonia in HIV-infected individuals. Conversely, observational data do not support corticosteroid use for influenza or fungal infections. In CAP complicated by septic shock or ARDS, corticosteroid use is endorsed by recent guidelines; however, the recommended timing, dosage, and duration vary. Although combination therapy with hydrocortisone and fludrocortisone is a potential option, further direct evidence is needed. Biomarkers such as C-reactive protein and, in the near future, insights into corticosteroid-related immune repair mechanisms in COVID-19 may aid in identifying corticosteroid-responsive phenotypes.

## Introduction

Pneumonia is an inflammatory disease of the lung parenchyma, most commonly caused by pathogenic microorganisms such as bacteria, viruses, and fungi. However, noninfectious forms of pneumonia resulting from autoimmune or allergic mechanisms also occur, and corticosteroid therapy is often indicated in such cases. This review focuses on the use of corticosteroids in the treatment of infectious pneumonia. 

Despite advances in treatment and expanded standard care, pneumonia remains a leading cause of mortality, especially among the elderly. It frequently contributes to the development of septic shock and acute respiratory distress syndrome (ARDS), both of which carry high mortality rates and often require intensive care.

In recent years, major international and regional clinical practice guidelines for the management of pneumonia and related conditions have been revised. For pneumonia, updated guidelines were released by European authorities in 2023, by Japan in 2024, and by France in 2025 [1–3]. Regarding sepsis, the most recent international guidance remains the 2021 Surviving Sepsis Campaign Guidelines (SSCG), whereas the updated Japanese guidelines (J-SSCG) were published in 2024 [4, 5]. For ARDS, revised guidelines were published by Japan in 2021, Europe in 2023, and the United States in 2024 [6–8]. In critically ill patients, relative adrenal insufficiency, termed critical illness-related corticosteroid insufficiency (CIRCI), is a recognized condition, and the latest CIRCI guideline, which addresses sepsis, ARDS, and CAP, was published in 2024 [9]. A comprehensive comparison of these guidelines is essential to elucidate the current strengths and limitations of the available evidence.

Additionally, extensive immunological and genetic research conducted during the SARS-CoV-2 infectious disease (COVID-19) pandemic has advanced our understanding of the pathophysiology of pneumonia, sepsis, and ARDS as well as the mechanisms underlying the efficacy of dexamethasone (DEX). This review also explores the immunological characteristics of these conditions and their modulation by corticosteroids, which may aid in identifying corticosteroid-responsive phenotypes.

### Causes, classification, and standard treatment of pneumonia

Infectious pneumonia is caused by the proliferation of pathogens within the lung parenchyma, leading to the infiltration of inflammatory cells, particularly neutrophils, and edematous changes. This inflammatory response is mediated by proinflammatory cytokines and chemokines and, in severe cases, can progress to septic shock or ARDS.

Pneumonia is classified into three main categories based on the setting of disease onset: community-acquired pneumonia (CAP), hospital-acquired pneumonia, which includes ventilator-associated pneumonia, and healthcare-associated pneumonia. Aspiration pneumonia, commonly noted in patients with dysphagia, can occur within any of these categories. These classifications differ in terms of causative pathogens, antimicrobial strategies, and clinical outcomes. Although antimicrobial therapy remains the cornerstone of treatment across all types, additional supportive care and adjunctive therapies, such as corticosteroids, are often necessary, especially in cases of severe CAP.

The definitions of severe CAP are summarized in Table 1. The most widely accepted criteria are those established by the 2007 Infectious Diseases Society of America (IDSA)/American Thoracic Society (ATS) consensus guidelines, which were reaffirmed in the updated 2019 ATS/IDSA guidelines on the management of CAP in adults [1]. Additionally, the inclusion and exclusion criteria used in the CAPE COD randomized controlled trial (RCT), which demonstrated the efficacy of corticosteroids in CAP, may provide further clinical insight (Table 2).

**Table 1:** Definitions of severe community-acquired pneumonia (CAP) in recent guidelines

Guidelines	CAP guidelines				CIRCI guidelines 2024
	American ATS/IDSA 2019	European ERS/ESICM/ESCMID/ALAT 2023	Japanese JRS 2024	French SPILF/SPLF 2025	
Definition	2007 IDSA/ATS criteria: ≥ 1 Major criteria • Septic shock with need for vasopressors • Respiratory failure requiring mechanical ventilation ≥ 3 Minor criteria • Respiratory rate > 30 breaths/min • PaO_2_/FIO_2_ ratio < 250 • Multilobar infiltrates • Confusion/disorientation • Uremia (BUN > 20 mg/dl) • Leukopenia (WBC < 4,000 cells/ml) • Thrombocytopenia (platelet count < 100,000 cells/ml) • Hypothermia (core temperature < 36 °C) • Hypotension requiring aggressive fluid resuscitation	ICU admission	A-DROP score • A (Age): Male ≥ 70 years old, Female ≥ 75 years old • D (Dehydration): BUN ≥ 21 mg/dl or signs of dehydration • R (Respiration): SpO_2_ ≤ 90% (PaO_2_ ≤ 60 Torr) • O (dysOrientation): due to pneumonia • P (systolic blood Pressure): ≤ 90 mmHg Severe: 3 of above Critical: ≥ 4 of above or with shock	ATS/IDSA 2007	List of • ATS/IDSA 2007 criteria • CAPE COD inclusion criteria [17] • Risk stratification scores: PSI class IV or V, etc.

*ATS* American Thoracic Society, *IDSA* Infectious Disease Society of America, *ERS* European Respiratory Society, *ESICM* European Society of Intensive Care Medicine, *ESCMID* European Society of Clinical Microbiology and Infectious Diseases, *ALAT* Latin American Thoracic Association, *JRS* Japanese Respiratory Society, *SPLIF* French Infectious Disease Society, *SPLF* French-Speaking Society of Respiratory Disease, *CIRCI* critical illness-related corticosteroid insufficiency, *BUN* blood urea nitrogen, *ICU* intensive care unit, *WBC* white blood cell, *PSI* pneumonia severity index

**Table 2:** Major differences among three recent randomized controlled trials in severe community-acquired pneumonia (CAP)

Trial name		ESCAPe (Meduri, et al.) [18]	CAPE COD (Dequin PF, et al.) [17]	REMAP-CAP (Angus DC, et al.) [20]
Trial design		Double-blind, randomized, placebo-controlled trial	Double-blind, randomized, placebo-controlled trial	Open-label, randomized, controlled trial
Hospitals		42 Veterans Affairs Medical Centers ICU 584 adults with severe CAP/HCAP	31 French Center ICU/Intermediate beds 800 adults with CAP	101 sites in 18 countries, 658 adults with CAP (455 were randomized to hydrocortisone or control at the 70 sites)
Sex: Male		96.4%	69.4%	60.8%
Inclusion criteria: Definition of severity		Including sepsis signs and nonpulmonary organ dysfunction ≥ 1 Major Criteria 1. Use of invasive or noninvasive mechanical ventilation 2. Vasopressors for shock despite adequate fluid resuscitation 3. Arterial pH < 7.30 OR ≥ 3 Minor Criteria 1. New onset of confusion or disorientation 2. Hypothermia (core temperature ≤ 36°C) 3. Respiratory rate ≥ 30 breaths/min 4. Hypotension requiring aggressive fluid resuscitation 5. Uremia (BUN ≥ 20 mg/dl) 6. PaO_2_: FIO_2_ ratio ≤ 250 OR SaO_2_:FIO_2_ ratio ≤ 250 7. Leukopenia (WBC count < 4000 cells/mm^3^) 8. Platelet count < 100,000 cells/mm^3^ OR > 400,000 cells/mm^3^ 9. Multilobar infiltrates	Primarily hypoxemia and acute respiratory failure Severity defined by at least one of the following: • Pneumonia Severity Index (PSI) > 130 (class V) • Patient placed on mechanical ventilation (invasive or not) for acute respiratory failure, with a PEEP level ≥ 5 cm H_2_O • Patient treated by high-flow oxygen therapy with a FIO_2_ ≥ 50% and PaO_2_:FIO_2_ ratio < 300 • Patient treated by oxygen therapy with a partial rebreathing-mask with a reservoir bag, provided that the PaO_2_ is less than the preset value corresponding to each inhaled oxygen flow rate	ICU admission and receiving organ support with one or more of: a. Non-invasive or invasive ventilatory support b. Receiving infusion of vasopressor or inotropes or both
Exclusion criteria		• Noradrenalin ≥ 0.3 μg/kg/min • CVP ≥ 8 (≥ 12 during ventilation) mmHg	• Influenza • Septic shock • Post-obstructive pneumonia	• HCAP • COVID-19
Corticosteroids	Timing	within 96 h	within 24 h	Enrolled within 24 h after ICU admission
	Dosage	mPSL 40 mg/day	Hydrocortisone 200 mg/day	Hydrocortisone 50 mg/6 h
	Duration	7 days, followed by tapering to 20 days	Either of the following depending on the patient’s response: • 4 days, followed by tapering to 8 days • 7 days, followed by tapering to 14 days	7 days and no tapering
Primary endpoint		60-day all-cause mortality: 16% in the intervention group vs. 18% in the control group	28-day all-cause mortality: 6.2% in the intervention group vs. 11.9% in the control group	90-day all-cause mortality: 15% in the intervention group (n = 536) vs. 9.8% in the control group (n = 122)

The key differences among three RCTs are summarized

*ICU* intensive care unit, *CAP* community-acquired pneumonia, *HCAP* healthcare-associated pneumonia, *BUN* blood urea nitrogen, *mPSL* methylprednisolone, *PEEP* positive end-expiratory pressure, *CVP* central venous pressure

### Secretion and functions of corticosteroids

Corticosteroids are classified into two main types: glucocorticoids and mineralocorticoids. Under physiological conditions, the adrenal glands in adults secrete approximately 5–11 mg of cortisol per day (equivalent to 20–30 mg of hydrocortisone) and 50–200 μg of aldosterone per day. In addition to their baseline physiological roles, both hormones are essential in mediating the body’s response to stress. Notably, their production is stimulated by overlapping signals, and their biosynthetic pathways partially converge. This overlap suggests the possibility of a relative deficiency of both hormones in critically ill patients [10].

Glucocorticoids regulate the transcription of a wide range of target genes, including those encoding proinflammatory and anti-inflammatory cytokines, adhesion molecules, complement components, and enzymes involved in arachidonic acid metabolism [11]. In addition to these genomic effects, glucocorticoids exert rapid nongenomic actions, including immunomodulatory effects and intracellular calcium regulation, through interactions with various cell surface receptors [12].

Apart from their well-established role in electrolyte regulation, mineralocorticoids are also involved in various immune responses [11]. Aldosterone, the primary mineralocorticoid, exerts rapid nongenomic effects, such as inducing vascular smooth muscle contraction [13]. In animal models of sepsis, aldosterone has been shown to restore endothelin-1 receptor expression, enhance catecholamine responsiveness, and improve survival outcomes [10]. Moreover, mineralocorticoids exert physiological effects on multiple organs, including the brain, heart, and kidneys [10].

In studies using lethal animal models, glucocorticoids, but not mineralocorticoids, were found to improve survival in a *Staphylococcus aureus*-induced pneumonia model [14, 15]. In contrast, both glucocorticoids and mineralocorticoids improved survival in a model of *S. aureus*-induced septic shock [16]. The potential effects of simultaneous administration of both hormones have not yet been evaluated. However, it is important to note that the findings from animal studies may not be directly applicable to humans.

### Current evidence and guideline recommendations for corticosteroid use in CAP

Two major RCTs investigating corticosteroid use in severe CAP were published in 2022 and 2023: the ESCAPe and CAPE COD trials, respectively [17, 18]. The ESCAPe trial found no significant difference in 60-day mortality (adjusted odds ratio [OR], 0.90; 95% confidence interval [CI] 0.57–1.40), whereas the CAPE COD trial demonstrated a significant reduction in 28-day mortality (hazard ratio, 0.59; 95% CI 0.40–0.86). Key differences between the two trials included the proportion of male participants, inclusion and exclusion criteria, and the corticosteroid regimen used (Table 2). In the ESCAPe trial, participants received 40 mg of methylprednisolone (mPSL) daily for a fixed duration of 7 days, whereas in the CAPE COD trial, participants received hydrocortisone at a total daily dose of 200 mg, administered for 4 or 7 days depending on their clinical response. Several systematic reviews (SRs) have been published to complement these findings, with most supporting the efficacy of corticosteroids in the treatment of severe CAP [19].

In 2025, a new RCT (REMAP-CAP) was published that evaluated the use of hydrocortisone 200 mg daily for a fixed duration of 7 days in patients with CAP [20]. The study did not demonstrate a significant reduction in 90-day mortality (adjusted OR, 1.56; 95% credible interval [CrI] 0.80–3.31). Key study characteristics and outcomes are summarized in Table 2. Mortality outcomes were analyzed across four patient subgroups (categorized by the presence or absence of influenza and shock), but no statistically significant differences were observed in any group. Notably, the study was affected by an uneven randomization process caused by a miscoding error, and enrollment was halted due to futility, potentially compromising the study’s statistical power.

Table 3 summarizes the current recommendations for corticosteroid therapy in recent pneumonia guidelines, including clinical questions related to its use. With the exception of the outdated 2019 ATS/IDSA guidelines, most recent guidelines generally recommend corticosteroids for selected cases. However, the strength of these recommendations varies across guidelines. Notably, the most recent guidelines do not reference the 2025 REMAP-CAP trial, which may influence future updates. At present, corticosteroid therapy should be tailored to the severity of CAP, with particular consideration given to the degree of hypoxemia and respiratory failure. In eligible patients, early administration (ideally within 24 h) is advised. Therapy may be discontinued early if the patient demonstrates a favorable clinical response. There is currently insufficient evidence to support corticosteroid use in aspiration pneumonia, which is explicitly excluded as an indication in the French guidelines. Corticosteroids are generally not recommended in such cases, even when acute respiratory failure following gastric aspiration suggests chemical pneumonitis as the predominant pathophysiology [21].

**Table 3:** Recommendations for corticosteroid use in the guidelines for community-acquired pneumonia (CAP)

		CAP guidelines				CIRCI guidelines 2024
		American ATS/IDSA 2019	European ERS/ESICM/ALAT 2023	Japanese JRS 2024	French SPILF/SPLF 2025	
Nonsevere CAP		E		D	E	–
Severe CAP	Recommendation	D When in shock, follow SSCG	B • When in shock • Not applicable to patients with viral pneumonia (influenza, SARS, MERS)	B	A • Except in cases of myelosuppression, aspiration pneumonia, or influenza	A For bacterial CAP
	Timing	–	–	–	within 24 h	• Regimen 1, 2, and 4: - • Regimen 3: within 36 h
	Dosage	–	mPSL 0.5 mg/kg/12 h	1. Hydrocortisone 200–300 mg/day 2. PSL 20–50 mg/day 3. mPSL 1 mg/kg/day 4. DEX 5–6 mg/day	Hydrocortisone 200 mg/day	1. Hydrocortisone 200 mg bolus, followed by 10 mg/h 2. Hydrocortisone 200 mg/day 3. mPSL 0.5 mg/kg/12 h 4. mPSL 40 mg/day
	Duration	–	5 days	3–7 days	Either of the following depending on the patient’s response: • 4 days, followed by tapering to 8 days • 7 days, followed by tapering to 14 days	1. 7 days 2. 4 or 8 days, followed by tapering to 8 or 14 days, discontinued on ICU discharge 3. 7 days 4. 7 days, followed by tapering to 20 days

A summary of recommendations for corticosteroid use in major pneumonia guidelines is provided

A, strongly recommended; B, weekly/conditionally recommended; C, expert opinion/research recommendation; D, weekly/conditionally not recommended; E, strongly not recommended; -, no recommendation

*ATS* American Thoracic Society, *IDSA* Infectious Disease Society of America, *ERS* European Respiratory Society, *ESICM* European Society of Intensive Care Medicine, *JRS* Japanese Respiratory Society, *SPLIF* French Infectious Disease Society, *SPLF* French-Speaking Society of Respiratory Disease, *CIRCI* critical illness-related corticosteroid insufficiency, *SARS* severe acute respiratory syndrome, *MERS* middle east respiratory syndrome, *mPSL* methylprednisolone, *PSL* prednisolone, *DEX* dexamethasone

### Evidence for corticosteroid use in nonbacterial pneumonia

The abovementioned recommendations regarding corticosteroid use in CAP are primarily based on RCTs involving patients with bacterial pneumonia. As a result, these recommendations may not be directly applicable to CAP caused by nonbacterial pathogens. This section reviews the current evidence and guideline recommendations for the use of corticosteroids in pneumonia caused by nonbacterial microorganisms.

During the ongoing COVID-19 pandemic, numerous clinical studies, including RCTs, have investigated the use of corticosteroids. DEX 6 mg daily has been established as the standard of care for patients requiring oxygen support, such as high-flow nasal cannula. However, the more recent RECOVERY trial found that a higher dose, DEX 20 mg daily, did not reduce mortality in hypoxic hospitalized patients requiring ventilatory support and may even be harmful in those not requiring such support [22, 23]. The NIH guidelines now recommend the use of additional immunomodulators, rather than increasing the corticosteroid dose, in severe or progressive cases [24]. Furthermore, a recent large retrospective analysis demonstrated that corticosteroids were effective in patients aged ≥ 60 years with severe disease, low lymphocyte counts, elevated C-reactive protein (CRP) levels, and the need for respiratory support [25]. These findings may help guide individualized corticosteroid use in patients with COVID-19.

To date, no RCTs have evaluated the use of corticosteroids in influenza-related pneumonia. However, multiple observational studies and corresponding SRs indicate that corticosteroid use may be harmful in this setting [26–28]. Consequently, influenza pneumonia is excluded from corticosteroid treatment recommendations in the French guidelines, and corticosteroid therapy is generally not advised. However, it is worth noting that these studies predominantly involved higher corticosteroid doses than those used in CAP trials, and data on low-dose regimens (≤ 40 mg/day mPSL equivalent) remain limited [27].

For *Pneumocystis jirovecii* pneumonia (PJP) in HIV-infected patients, the NIH Guidelines for HIV-Associated Opportunistic Infections recommend corticosteroid therapy when the partial pressure of arterial oxygen (PaO_2_) is < 70 mmHg or the alveolar–arterial oxygen gradient is ≥ 35 mmHg [29]. The suggested regimen is prednisolone 40 mg twice daily for 5 days. This recommendation is based on RCTs conducted in the 1990s and subsequent SRs. Conversely, there is a lack of RCTs evaluating corticosteroid therapy for PJP in non-HIV immunocompromised patients. However, observational studies in patients with acute hypoxemia and respiratory failure indicate that a similar corticosteroid regimen may be beneficial [30]. For other types of fungal pneumonia, RCT evidence is also lacking. A recent SR indicates that corticosteroid use in invasive fungal infections may be associated with worse outcomes [31]. Therefore, as emphasized in the ATS statement, corticosteroid therapy in these cases should be carefully individualized [32].

### Corticosteroids for septic shock and ARDS in CAP

A retrospective study from the early 1990s reported that 48% of patients with CAP developed severe sepsis, and 4.5% progressed to septic shock [33]. More recently, a prospective study found that approximately 38% of patients with CAP were complicated by severe sepsis [34]. 

Two large RCTs for septic shock, ADRENAL and APROCCHSS, were published in 2018, with the former showing no significant improvement in 90-day mortality (28.8% in the hydrocortisone group vs. 27.9% in the placebo group; OR, 0.95; 95% CI 0.82–1.10; *p* = 0.50), whereas the latter, which included more severely ill patients, demonstrated a significant reduction in 90-day mortality (43.0% in the hydrocortisone-plus-fludrocortisone group vs. 49.1% in the placebo group; relative risk [RR], 0.88; 95% CI 0.78–0.99; *p* = 0.03) [35, 36]. Most recent SRs, including those conducted during the guideline update process, have shown significant improvements in mortality, and the latest sepsis guidelines generally recommend low-dose corticosteroids, typically hydrocortisone 200 mg daily, for septic shock (Table 4).

**Table 4:** Recommendations for corticosteroid use in sepsis and CIRCI guidelines

Guidelines		Sepsis guidelines		CIRCI guidelines 2024
		International (SSCG 2021) SCCM/ESICM	Japanese (J-SSCG 2024) JSICM/JAAM	
Low-dose corticosteroids	Recommendation	B For septic shock	B For septic shock	B For septic shock
	Timing	–	–	–
	Dosage	Hydrocortisone 200 mg/day (50 mg/6 h or continuous infusion)	Hydrocortisone 200–300 mg/day	Hydrocortisone 200 mg/day ± Fludrocortisone 50 µg/day
	Duration	–	–	7 days or until ICU discharge

A summary of recommendations for corticosteroid use in various sepsis and CIRCI guidelines is provided

A, strongly recommended; B, weakly/conditionally recommended; C, expert opinion/research recommendation; D, weakly/conditionally not recommended; E, strongly not recommended; -, no recommendation

*SSCG* Surviving Sepsis Campaign Guidelines, *SCCM* Society of Critical Care Medicine, *ESICM* European Society of Intensive Care Medicine, *JSICM* Japanese Society of Intensive Care Medicine, *JAAM* Japanese Association for Acute Medicine, CIRCI critical illness-related corticosteroid insufficiency

Regarding ARDS, a large retrospective analysis reported that 2.7% of patients with CAP developed ARDS [37]. The most recent RCT, DEXA-ARDS, demonstrated a significant survival benefit with DEX administered at 20 mg daily for 5 days, followed by a tapering regimen [38]. Most SRs, whether independent or conducted as part of guideline development, have also reported a survival benefit. Accordingly, recent guidelines that address the clinical question of corticosteroid therapy recommend corticosteroids for the management of ARDS (Table 5) [6, 8, 39–41].

**Table 5:** Recommendations for corticosteroid use in ARDS and CIRCI guidelines

Guidelines	ARDS guidelines					CIRCI guidelines 2024
	American ATS 2024	Japanese JSICM/JRS 2021	UK FICM/ICS 2018	Scandinavian 2016	Korean KSCCM/KATRD 2016	
Recommendation	B	A	C	E	D	B
Timing	–	Early*				Regimen 1. Early (≤ 24 h) Regimen 2. Early (≤ 72 h) Regimen 3. Unresolving ARDS (7–21 days)
Dosage	–	mPSL 1–2 mg/kg**				1. DEX 20 mg/day 2. mPSL 1 mg/kg 3. mPSL 2 mg/kg
Duration	–	≥ 7 days*				1. 5 days, followed by 10 mg for 5 days until extubation 2. 14 days, followed by tapering to 28 days If extubated before day 14, then advance to 0.5 mg/kg/day 3. 7 days, followed by tapering to 32 days If extubated before day 14, then advance to 1 mg/kg/day

A summary of recommendations for corticosteroids in various ARDS and CIRCI guidelines is provided

A, strongly recommended; B, weakly/conditionally recommended; C, expert opinion/research recommendation; D, weakly/conditionally not recommended; E, strongly not recommended; -, no recommendation

^*^suggested in subgroup analysis, **cited as a definition of low-dose corticosteroid therapy

*ATS* American Thoracic Society, *JSICM* Japanese Society of Intensive Care Medicine, *JRS* Japanese Respiratory Society, *FICM* Faculty of Intensive Care Medicine, *ICS* Intensive Care Society, *KSCCM* Korean Society of Critical Care Medicine, *KATRD* Korean Academy of Tuberculosis and Respiratory Diseases, *CIRCI* critical illness-related corticosteroid insufficiency

The recently revised CIRCI guideline included several subanalyses, which suggest that among corticosteroids, DEX, early initiation of therapy, a daily dose of < 88 mg mPSL equivalent, and use in ARDS unrelated to COVID-19 are associated with improved outcomes. These findings may help guide individualized decisions regarding corticosteroid therapy [9].

### Potential benefit of adding mineralocorticoid to glucocorticoid

As noted above, mineralocorticoids possess several vasoactive and immunoregulatory properties, and a relative deficiency of both mineralocorticoids and glucocorticoids may occur under critical conditions. Annane et al. demonstrated the efficacy of combination therapy with hydrocortisone and fludrocortisone for septic shock in both the initial French trial and the subsequent APROCCHSS trial [36, 42]. During the development of the J-SSCG 2020, an SR was conducted, including three studies (*n* = 2,050), which supported a weak recommendation in favor of combination therapy (RR, 0.88; 95% CI 0.78–0.99) [43]. However, this clinical question was omitted in the more streamlined J-SSCG 2024 [4]. A recent individual patient data meta-analysis of 24 trials (*n* = 8,528), including 17 trials with individual-level data (*n* = 5,929), reported an RR of 0.86 (95% CI 0.79–0.92) for 90-day mortality with combination therapy, compared to 0.96 (95% CI 0.82–1.12) for hydrocortisone alone [44]. Furthermore, a network meta-analysis of 17 studies (*n* = 7,688) showed that combination therapy was associated with a 12% lower risk of mortality compared to hydrocortisone alone (RR, 0.88; 95% CrI 0.74–1.03) [45]. However, it should be noted that this comparison was largely based on indirect evidence. Importantly, these studies did not report an increased risk of adverse events with combination therapy. Additionally, a large cohort study (*n* = 88,275) found that hospital mortality was significantly lower in the combination therapy group than in the hydrocortisone-alone group (adjusted absolute risk difference, –3.7%; 95% CI −4.2–−3.1%; *p* < 0.001) [46].

Regarding CAP, a recent post hoc analysis of the APROCCHSS trial in patients with CAP and septic shock demonstrated that combination therapy significantly reduced mortality in patients with CAP (OR, 0.60; 95% CI 0.43–0.83) but not in those without CAP (OR, 0.95; 95% CI 0.70–1.29) [47]. Similarly, a large cohort study of patients with septic shock, including 32,713 patients with CAP and 55,562 without CAP, showed that combination therapy was associated with reduced mortality in both subgroups: RR 0.94 (95% CI 0.93–0.96) in patients with CAP and RR 0.90 (95% CI 0.89–0.91) in those without CAP [48]. In the former study, a subgroup analysis of patients with ARDS (*n* = 648) suggested that combination therapy was associated with lower mortality in patients with ARDS (OR, 0.72; 95% CI 0.53–0.98) but not in those without ARDS (OR, 0.85; 95% CI 0.61–1.20). However, the authors noted that this subanalysis was likely underpowered [47].

Despite the aforementioned indirect evidence supporting combination therapy in septic shock specifically caused by CAP, no RCTs have directly compared hydrocortisone plus fludrocortisone with hydrocortisone monotherapy and demonstrated clear efficacy. In the COIITSS trial, a two-by-two multifactorial open-label study comparing continuous intravenous insulin infusion with conventional insulin therapy as well as hydrocortisone plus fludrocortisone with hydrocortisone monotherapy in patients with septic shock and multiple organ dysfunction, there was a 2.9% absolute reduction in hospital mortality in the combination therapy group (*n* = 245) compared to the hydrocortisone monotherapy group (*n* = 264) [49]; however, this trial was statistically underpowered. A recent open-label, phase II RCT investigating the dose–response relationship of fludrocortisone found no significant differences in the primary outcome of time to shock resolution or in other secondary outcomes [50]. Notably, the absolute 28-day mortality rates were progressively lower in the combination therapy groups—24%, 17%, 11%, and 11%—among patients treated with hydrocortisone 200 mg plus oral fludrocortisone at daily doses of 0, 50, 100, and 200 µg, respectively. This study also revealed high interindividual variability in plasma fludrocortisone levels following enteral administration, suggesting inconsistent absorption and metabolism among patients. In conclusion, although the addition of fludrocortisone to hydrocortisone may enhance treatment efficacy without increasing adverse events in patients with CAP complicated by septic shock and ARDS, further high-quality RCTs using optimized fludrocortisone dosing are necessary before strong recommendations can be endorsed by the majority of experts.

### Novel immune repair mechanisms of DEX in COVID-19 and the corticosteroid-responsive phenotype

During the ongoing COVID-19 pandemic, extensive research has advanced the understanding of disease pathophysiology, revealing both immunological similarities and differences between COVID-19, bacterial ARDS, and severe influenza. A study comparing humoral mediator profiles in patients with influenza and COVID-19 demonstrated that the expression levels of interleukin-2 (IL-2), a proliferation-inducing ligand (APRIL), soluble tumor necrosis factor receptors 1 and 2 (sTNF-R1 and sTNF-R2), surfactant protein D, and chemokine (C-X-C motif) ligand 17 (CXCL17) were characteristically elevated in influenza. In contrast, the expression levels of tumor necrosis factor-related weak inducer of apoptosis (TWEAK), thymic stromal lymphopoietin (TSLP), and matrix metalloproteinases 1 and 3 (MMP-1 and MMP-3) were specifically upregulated in COVID-19 [51]. These findings indicate that the kinetics and regulation of immune mediators vary by pathogen, resulting in distinct immunological phenotypes despite similar clinical presentations [51].

As the efficacy of DEX in moderate-to-severe COVID-19 has been well established, its underlying mechanisms of action have also been explored. A study comparing patients with bacterial ARDS and those with COVID-19-related ARDS revealed an increased presence of interferon (IFN)-activated neutrophils, particularly in patients with severe COVID-19 [52]. Following DEX administration, global transcriptional activity was suppressed, and expression of interleukin-1 receptor type 2 (IL-1R2) was upregulated in IFN-activated neutrophils. Additionally, the overall neutrophil count, especially IFN-stimulated neutrophils, was reduced, whereas the numbers of IL-1R2-positive neutrophils and immunosuppressive immature neutrophils increased in DEX-treated patients. Plasma proteomic analysis further demonstrated a reduction in the levels of proinflammatory proteins, including members of the S100A protein family, in patients receiving DEX [52].

In a subsequent study, DEX treatment was associated with reduced expression of genes involved in T cell activation, including TNF receptor superfamily member 4 (*TNFRSF4*) and interleukin-21 receptor (*IL21R*) [53]. The most pronounced transcriptional changes in the DEX-treated group were observed in neutrophils, notably with the downregulation of *S100A* protein family genes in both blood and tracheal aspirates. Additionally, gene expression related to major histocompatibility complex class II (MHC-II) signaling and selectin P ligand (SELPLG) signaling was significantly decreased in the lungs [53].

A more recent study analyzed blood and bronchoalveolar lavage (BAL) fluid cells in relation to clinical outcomes in DEX-treated patients and identified monocytes and alveolar macrophages as the primary target cells of DEX [54]. In DEX-treated survivors, gene expression of proinflammatory mediators, including S100A proteins, cytokines, and chemokines such as CXCL1, was significantly reduced. Additionally, reconstitution of MHC-II expression was observed in blood monocytes and BAL monocytes/macrophages [54]. Based on these findings, the authors proposed that whole blood transcriptome-based signatures may have potential for predicting responsiveness to DEX. These results suggest that the therapeutic effects of corticosteroids involve not only classical anti-inflammatory mechanisms, but also active immune repair processes.

A recent data-driven analysis of RCTs indicated that CRP levels above 204 mg/L may serve as a useful biomarker for identifying patients who are likely to benefit from corticosteroid therapy [55]. Similarly, the recently elucidated mechanisms underlying corticosteroid efficacy in COVID-19 may have practical clinical applications. Although further validation is needed, certain elevated mediators or specific immune cell subset alterations—observed in patients with COVID-19 and reversed by DEX treatment—may help identify corticosteroid-responsive phenotypes. In the near future, the development of benchtop assays capable of detecting corticosteroid responsiveness could enable personalized corticosteroid therapy for CAP and other inflammatory conditions.

### Concluding remarks

Recent clinical guidelines generally recommend the use of corticosteroids for severe bacterial CAP and CAP complicated by septic shock or ARDS. However, the optimal timing, dosage, and duration of corticosteroid therapy may vary depending on the presence and type of complications. For nonbacterial pneumonia, high-quality evidence supporting corticosteroid use is currently limited to patients with COVID-19 and HIV-associated PJP. In contrast, observational data do not support corticosteroid use for influenza or fungal infections. Combination therapy with hydrocortisone and fludrocortisone may be beneficial for patients with CAP complicated by septic shock and ARDS; however, further direct evidence, particularly regarding appropriate fludrocortisone dosing, is needed to support strong recommendations. CRP levels may serve as a useful clinical biomarker for guiding corticosteroid therapy, and in the near future, emerging insights into the immunomodulatory mechanisms of corticosteroids in COVID-19 may help identify corticosteroid-responsive phenotypes.

## Data Availability

No datasets were generated or analyzed during the current study.
